# Epidemiology and clinical spectrum of pediatric patients with human immunodeficiency virus (HIV) infection: A 15 years’ experience

**DOI:** 10.12669/pjms.39.3.6710

**Published:** 2023

**Authors:** Naila Bashir, Nighat Haider, Ana Farooq, Mulazim Hussain

**Affiliations:** 1 Dr. Naila Bashir, Medical Officer, Incharge HIV Clinic, Pakistan Institute of Medical Sciences (PIMS), Islamabad, Pakistan; 2 Dr. Nighat Haider, Assistant Professor, Head of Pediatric Infectious Diseases Unit, Shaheed Zulfiqar Ali Bhutto Medical University, Pakistan Institute of Medical Sciences (PIMS), Islamabad, Pakistan; 3 Dr. Ana Farooq, Senior Registrar, Pakistan Institute of Medical Sciences (PIMS), Islamabad, Pakistan; 4 Dr. Mulazim Hussain, Assistant Professor, Pakistan Institute of Medical Sciences (PIMS), Islamabad, Pakistan

**Keywords:** Human immunodeficiency virus, Children, Clinical presentation, Co-infection, Mode of transmission, Pediatric population

## Abstract

**Objective:**

To view the different patterns of presentation of HIV in pediatric population along with mode of transmission and associated co infections and co morbidities

**Methods:**

It was a retrospective study conducted at Pakistan Institute of Medical Sciences, Islamabad, in which we evaluated the records of pediatric patients diagnosed with HIV from 2005 to 2020. All the data like age, gender, area, presenting complaints, examination findings at the time of diagnosis, mode of transmission, co infection and co morbidities were recorded. Descriptive analysis was done to calculate frequencies and means of the variables. SPSS 20 was used for data analysis.

**Results:**

Ninety four participants were evaluated with male to female ratio as 1.8:1 and mean age of 5.2 years. Majority of patients (44%) were below 4 years. Fever (55%) was the most reported symptom followed by cough (39%), diarrhoea (29%), pallor (27%), shortness of breath (26%), weight loss (23%) and failure to thrive (22%). Co infection with TB was present in (16%). Eight (9%) patients were thalassaemic. Mother to child transmission (60%) was the commonest mode of transmission followed by blood transfusion (23%) and parenteral transmission (6%).

**Conclusion:**

In children HIV is more prevalent in males especially under 4 years with fever, cough, diarrhea and pallor being the common symptoms at presentation. Tuberculosis is the commonest co infection as we are endemic for TB and mother to child transmission is the commonest mode of transmission as there was no outbreak in our area.

## INTRODUCTION

Human immunodeficiency virus (HIV) infection is one of the diseases with inimical medical issues along with associated social taboos to be taken care of by the family. According to World Health Organization (WHO) around 38 million people are living with HIV/AIDS,^1^ 1.8 million (4.7%) of these are children under 15 years of age. While looking at Pakistan’s data, according to UNAIDS data sheet, at the end of 2019 around 190 000 people were living with HIV, out of which 6,100 (3.2%) were children zero to fourteen years which is less than world’s percentage of children with HIV.^2^

Most children present before two years of age as vertical transmission is commonest route of pediatric HIV acquisition.^3^ The earlier one acquires the infection, more fatal will be the progress of disease and hence the outcome. The pediatric patients with HIV infection usually present with recurrent chest infections, chronic diarrhea, cutaneous manifestation, failure to thrive, lymphadenopathy, visceromegaly and thrombocytopenia or pancytopenia.^3^ HIV infection is also an important contributing factor to the increase incidence of severe malnutrition and this lethal combination results in high mortality among this already vulnerable population. Severe wasting is a well-known feature of pediatric HIV infection with decrease response to therapy and adverse clinical outcome.^4^ HIV infection may also predispose to bacterial, fungal and viral infections. The opportunistic infections like tuberculosis, candidiasis, herpes, varicella, Ebstein Barr Virus (EBV), Cytomegalovirus (CMV) and Pneumocystis jirovecii are the major cause of death in these patients. HIV encephalopathy, associated with loss of developmental milestones, cerebral atrophy, and acquired motor deficit have also been reported.^5^

By studying the patterns of clinical presentation of HIV in pediatric population we will be able to understand the different ways of exhibition of disease more effectively, which will help us in early diagnosis of the disease and timely management of patients. More importantly study will help in viewing the pattern of presentation in Pakistani pediatric population.

## METHODS

All children under the age of 12 years, diagnose with human immunodeficiency virus (HIV) infection at Pakistan Institute of Medical Sciences, Islamabad, from 2005 to 2020 were included in the study. The patients who were positive by RT PCR or serology were considered to have disease. It was a retrospective study in which we evaluated the records of pediatric patients. The patients were included on the basis of complete medical record. We captured the relevant study information on predesigned structured proforma. All the clinical data like age, gender, ethnicity, presenting complaints, examination findings at the time of diagnosis, mode of transmission, any co infection and co morbidities were recorded as well. Complete blood count and WHO clinical stage at the time of diagnosis was also recorded. Informed consent was obtained from patients/ guardian and study was started after taking approval from ethical review board (F. 1-1/2015/ERB/SZABMU/641 dated 14-09-2020) Descriptive analysis was done to calculate frequencies and means of the variables. SPSS 20 was used for data analysis.

## RESULTS

In this study, 94 participants were recruited to explore the most prevalent clinical symptoms along with co-infections and co-morbidities. Of these, 61 (65%) were males and 33 (35%) were females with male to female ratio as 1.8:1 (Table-I). Independent sample t-test was calculated to explore the difference in prevalence of symptoms between males and females however the results indicated that no significant difference exist. The age range of the participants was from two months to 12 years with a mean age of five years (±4.12). Majority of participants belonged to age group of 0.02 to 3.11 years (44%) (Table-I).

**Table-I:** Baseline characteristics of the cases (N=94).

Variables	Categories	No of cases (%)
Age (years)	0.02-3.11	41 (44)
	4-7.11	21 (22)
	8-11.11	24 (26)
	12-16	8 (9)
	Mean (SD)	5.27 (4.12)
Gender	Male	61 (65)
	Female	33 (35)

While exploring the prevalence of clinical symptoms, a list of 24 indicators was generated based on the findings of clinician. The researcher intended to find symptoms which were most common. Thus, frequencies were calculated to explore the most reported clinical symptoms. It was found that 20 cases reported no clinical symptoms although some had a co-infection or co-morbidity. Around 13 cases reported at least one or two symptoms and 12 reported 3-4 symptoms. Highest number of symptoms reported was 10 but it was found in only one case. While exploring the prevalence of symptoms, Fever was the most reported symptom as found in 52(55%) of the cases followed by cough 37(39%) and diarrhoea 27(29%). Additionally, pallor 25(27%), shortness of breath 24(26%), weight loss 22(23%) and failure to thrive 21(22%) was also found (Table-II). The initial complete blood count showed in 63(67%) of the patients. On the initial complete blood count 49(52%) patients had pancytopenia while only 3(3%) patients had isolated thrombocytopenia. Out of 94 patients’ maximum patients 52 (55.3%) were in WHO Stage-3, followed by 31 (33%) patients in Stage-1, while 6(6.4%) were in Stage-2 and 5(5.3%) in Stage-4. It was seen that out of 94 cases, 32 reported co infections among which tuberculosis was the most prevalent 15(16%) followed by soft tissue infection 6(6%) (Table-III).

**Table-II:** Prevalence of Clinical Symptoms (n=94)

Variables	No of cases (%)
Fever	52 (55)
Cough	37 (39)
Diarrhea	27 (29)
Anemia	25 (27)
Shortness of Breath	24 (26)
Weight loss	22 (23)
Failure to thrive	21 (22)
Oral Thrush	18 (19)
Pallor	12 (13)
Fatigue	8 (8)
Vomiting	7 (7)
Cervical Lymphadenopathy	4 (4)
Abdominal Pain	3 (3)
Abdominal Distention	3 (3)
Poor Appetite	3 (3)
Hepatosplenomegaly	3 (3)
Ascites	2 (2)
Hepatomegaly	2 (2)
Mouth ulcers	1 (1)
Night Sweat	1 (1)
Body ache	1 (1)
Neuropathy	1 (1)
Jaundice	1 (1)

**Table-III:** Prevalence of Co Infection (n=32)

Variables	No of cases (%)
TB	15 (16)
Soft Tissue Infection	6 (6)
HCV	2 (2)
UTI	2 (2)
PCP	2 (2)
Axillary LN abscess	1 (1)
Candidiasis	1 (1)
Herpes zoster	1 (1)
HSV	1 (1)
Septic Arthritis	1 (1)

Furthermore, 11 cases reported comorbidities as well. Thalassemia was found in eight cases while two cases had Haemophilia and one reported with Von Willibrand disease. Most common mode of transmission was from Mother to Child 56 (60%) followed by blood transfusion 22 (23%) and parenteral transmission 6(6%). One patient got the infection by sexual abuse and in seven patients the mode of transmission was unknown.

Majority of patients were from Rawalpindi 18 (19%) followed by Gujarat 15 (16%), Islamabad 12 (13%), Azad Kashmir eight (9%), Jhelum four (4%), Battagram three (3%), Daira Ghazi Khan three (3%) and two each from Abbotabad, Attock, Bakhar, Mansehra, Peshawar, Sarghoda. One patient each from other nearby cities.

## DISCUSSION

Human immunodeficiency virus was thought to be exiguous in Pakistan especially in children but with time more cases are being reported. Looking at the data from 2005 to 2015, there is quick rise in number of patients from 8,360 to 45,990 i.e., 17.6% making it the highest in the history.^6^ As the number of infection in adults is rapidly increasing, the rate of HIV infection among children is also on the rise, which is estimated to be 54% over the past 13 years in Pakistan.^7^ Therefore it was important to know what are the common presenting complaints, mode of transmission and co infection in these children especially in our set up. HIV center was established at our institute in 2005 and we are looking after pediatric patients with HIV from last 16 years.

Out of total 94 participants the male to female ratio was 1.8:1. most common presenting symptoms was fever (55%) followed by cough (39%) and diarrhoea (29%). The majority of participants in our study were less than four years of age (44%) which is expected when most common mode of transmission is MTCT. Similar findings are described in other studies from Pakistan.^8^,^9^ While a study from South Africa assessing horizontal transmission of HIV in patients with uninfected mother reported median age of presentation to be 79 months.^10^

There is male predominance seen in HIV infection not only in our study where out of 94 cases 61(65%) were boys and 33(35%) were girls but other studies as well. In a study from Islamabad there were 63% boys and 37% girls while a study from Karachi showed almost the same results with 62% males and 38% females.^8^,^9^ Similar results are seen in Indian,^11^ and Nepali^12^ population. The possible reason for this may be because boys are given importance for seeking early medical consultation hence early and prompt diagnoses. This explanation though implies to our and Indian population but probably not to other communities. The difference in immune status and hormones may also play a role. Although a study from Oman^13^ showed slight female (54%) predominance.

Fever was the most common presenting symptoms (55%) in children in our study which is in consistent with the other studies from India and Nigeria where fever was the commonest symptom (53%) and (95.4%) respectively.^14^,^15^ Although in another study from Nigeria weight loss (62.5%), was more prevalent than prolonged fever (55.4%) followed by generalized lymphadenopathy (48.6%) and chronic cough (45.4%).^16^ Similarly a Pakistani study showed failure to thrive (70%) and persistent diarrhea (67%) being the commonest presenting features with fever only in 26% of Patients.^8^ The common symptoms vary in different studies. Sütçü et al.^3^ in their study from Turkey showed lymphadenopathy (54.5%) and recurrent respiratory tract infections (36.4%) to be the common presentations. In developed countries systemic and pulmonary symptoms are the presenting symptoms while in developing countries children usually presents with chronic diarrhea, pneumonia and severe acute malnutrition.^17^ HIV infection is one of the leading causes of malnourishment in children due to recurrent infections resulting in increased metabolic demand and anorexia. Forty percent of patients with HIV are malnourished and the concurrence of both the condition results in adverse outcome.^18^

We did not report any oncological conditions although there is 4-14 fold^19^ higher risk of developing cancer in pediatric patients with HIV infection. There may be miss diagnosis or the children might not have survived to the age to develop cancer.

Mother to child transmission (MTCT) is the commonest mode of transmission in children. It can occur during intrauterine life, during delivery or after the child birth. The risk of MTCT depends on the viral load, mode of delivery, use of antiretroviral drugs by the mother. In established maternal infections the risk of transmission via breastfeeding is 14% and if the mother has acute infection the risk rises to 29%. Formula feeding can reduce this risk of transmission to the newborn baby but this is challenging in developing countries like ours where other factors have to be taken into account as affordability of formulas may be an issue due to poverty and lack of breast feeding may lead to other severe infections like acute gastroenteritis and pneumonia. Hence in developing countries it is recommended to continue breastfeeding but mothers are provided with the antiretroviral therapy (ART) which reduces the risk of MTCT while ART given during pregnancy may reduce the risk of vertical transmission by 51% by decreasing the viral load. While to prevent transmission of HIV from mother to child during childbirth cesarean sections is the preferred mode of delivery.

In our study as well majority of the cases (60%) had MTCT. Our study spans over 15 years and there were no outbreak in our area over this duration. Although MTCT is the most common mode of transmission but it is observed that in out breaks usually the mode of transmission is unsafe injection practices and blood transfusion which is the cause of tremendous increase in number of pediatric patients with HIV in Pakistan. In last two decades around eight outbreaks have been reported. 20 In 2019 in outbreak of HIV at Rotedero Sindh, 763(82%) individuals were less than 16 years and 604(79%) were less than 5 years of age with only 11% having mothers with HIV.^21^ The major cause of spread in this and other out breaks is unethical practice of reusing contaminated needles for antibiotics and fluids by quacks and physicians and unscreened blood transfusions to several children. Our study also showed that 6 of our children had parental mode of transmission with history revealing having intravenous injections for antibiotics or fluids at some time in life before presenting with HIV to our center. As children are not injectable drug abusers, this node of transmission is very low in children as compare to adults.^22^,^23^

In 2019, 46 thalassemia patients were reported to have HIV in Karachi due to unsafe blood transfusions from unregistered sources with half of them co infected with hepatitis B.^24^ According to National Aids Control Program around 20-40% of blood is not screened for any transmittable diseases despite of the law for mandatory blood screening. Our study also revealed that unsafe blood transfusion practicing has been playing a major role in spread of the disease, with 22 cases (23%) having history of blood transfusion in the past. Out of these 22 patients 8 patients had thalassemia. There is also lack of awareness among the general population about safe blood transfusion. After an outbreak at Kot Imrana 96% of HIV patients were unaware of the modes of transmission of HIV infection where prevalence of HIV raised from 1.29% to 13.39% in just six months. 25

Tuberculosis is the commonest opportunistic infection in HIV infected individuals especially in the developing countries. HIV infection and TB aggravate each other. As HIV infection potentiates tuberculosis, tuberculosis also leads the progression of HIV infection to AIDS. The main factor of immunosuppression in HIV patients is the loss of CD4+ T cells, which contributes to the increased incidence of developing active TB. The patients with HIV can develop both primary TB and secondary TB either by reactivation or reinfection.^26^ Our study also showed that 16% of the cases were diagnosed with TB, and on their further workup tested positive for HIV. According to WHO guidelines it is recommended that every child diagnosed with TB should be screened for HIV and vice versa.

Our study also showed that 35% of the cases were from Punjab province mainly from Rawalpindi 18 (19%) and Gujarat 15 (16%) cities, 13% from the Islamabad and around 10% cases from various cities of Khyber Pakhtunkhwa province. Gujarat and Sargodha are two cities in Punjab with high HIV infection rate after large outbreak in these regions again due to malpractices by health care providers.^27^ In Pakistan there are many areas which are hubs of HIV infections like Sheikhupura, Lahore, Faisalabad, and around 50 villages nearby Sargodha and Mardan.^19^

HIV infection in children has a wide spectrum of clinical presentation which may vary according to the geographical territory like we did not report any child with recurrent parotitis, HIV encephalopathy or any malignancies. This study was very important as it helped us to know the presenting sign and symptoms, mode of transmission and comorbidities associated with HIV in our area.

We recommend to screen children with prolonged fever, chronic cough and diarrhea for HIV. Routine screening of all mothers for HIV during pregnancy should be done to prevent mother to child transmission which is the commonest mode. Blood products screening and strict rules for safe clinical practices should also be state policy to prevent the HIV outbreaks.

### Limitation:

The limitation of study is that it is a single center study and we feel that at the beginning many co infections may have been missed because of limited resources at that time like PCP.

## CONCLUSION

In children HIV is more prevalent in males especially under 4 years with fever, cough, diarrhea and pallor being the common symptoms at presentation. Tuberculosis is the commonest co infection as we are endemic for TB and mother to child transmission is the commonest mode of transmission as there was no outbreak in our area.

### Authors’ contribution:

**NB:** Helped in collection of data, conception of the study and participated in its design. She also helped to draft the manuscript.

**NH:** Participated in the design of the study and performed the statistical analysis along with drafting of manuscript. She is responsible and accountable for the accuracy or integrity of the work.

**AF:** Also help in collection of data and its interpretation along with input in drafting of manuscript

**MH:** Critically reviewed the manuscript, and approved the final manuscript as submitted.
